# Suitability and Trueness of the Removable Partial Denture Framework Fabricating by Polyether Ether Ketone with CAD-CAM Technology

**DOI:** 10.3390/polym16081119

**Published:** 2024-04-17

**Authors:** Kening Zhao, Su Wu, Chao Qian, Jian Sun

**Affiliations:** 1 Department of Prosthodontics, Shanghai Ninth People’s Hospital, Shanghai Jiao Tong University School of Medicine, Shanghai 200011, China; corallll@sjtu.edu.cn (K.Z.); wusu_13167168596@163.com (S.W.); 2 College of Stomatology, Shanghai Jiao Tong University, Shanghai 200011, China; 3 National Center for Stomatology, National Clinical Research Center for Oral Diseases, Shanghai Key Laboratory of Stomatology, Shanghai Research Institute of Stomatology, Shanghai 200011, China; 4 Department of Dentistry, Shanghai East Hospital, Tongji University School of Medicine, Shanghai 200120, China

**Keywords:** polyether ether ketone, suitability, trueness, removable partial denture, CAD-CAM

## Abstract

The object of the study was to evaluate the suitability and trueness of the removable partial denture (RPD) framework fabricated by polyether ether ketone (PEEK) with the CAD-CAM technology in vitro. Four different types of dentition defects were selected. In each type, five PEEK RPD frameworks were fabricated by the CAD-CAM technology, while five Co-Cr RPD frameworks were made by traditional casting. The suitability of the framework was evaluated by silicone rubber film slice measurement and the three-dimensional image overlay method. The trueness of the PEEK framework was detected by the three-dimensional image overlay method. Data were statistically analyzed with the use of an independent samples *t*-test (α = 0.05). The suitability values by silicone rubber film slice measurement of the PEEK group were lower than those of the Co-Cr group in four types, with the differences indicating statistical significance (*p* < 0.05) in type one, type two, and type four. The suitability values using the three-dimensional image overlay method showed no statistical differences (*p* > 0.05) between the two groups in four types. The trueness values of the PEEK group were within the allowable range of clinical error. The suitability and trueness of the PEEK RPD framework fabricated by CAD-CAM technology met the requirements of the clinical prosthesis.

## 1. Introduction

Despite the widespread use of implant dentures, removable partial dentures (RPDs) remain the most commonly used method for repairing dental defects in clinical practice. Removable partial dentures are favored in prosthetics because of their wide application range, simple manufacturing process, relatively low cost, simple post-repair, and the fact that patients can remove and put them on themselves [1,2]. With technological advancements, the requirements of removable partial dentures are not only limited to the restoration of function but also focus on beauty and comfort [3]. Nonetheless, the problem of significant differences in the resistance properties between plastics and metals, along with stress concentration at the joint, cannot be ignored [4].

Polyether ether ketone (PEEK) is a high polymer composed of one ketone bond and two ether bonds in the main chain structure, making it a unique polymer material. Due to its fine biocompatibility, mechanical properties, and stress conductivity, several researches have used PEEK as a restorative material in the field of dental implants [5]. Recent advancements have seen PEEK’s application broaden to include both fixed and removable partial dentures [6,7,8]. A study by Costa et al. indicated PEEK materials were used to make prosthetics for patients of maxillary defects with satisfactory restoration results [9]. Chen et al. found that the PEEK RPD frameworks induced less stress on abutment teeth and periodontal membranes than metal ones, suggesting PEEK could be considered a viable option for denture restoration in patients with periodontal disease [10]. Therefore, PEEK can not only improve the aesthetic appeal of removable partial denture restoration but can also eliminate allergic reactions and other discomfort caused by metal materials in traditional removable partial dentures [11,12].

With the wide use of digital technology in prosthodontics, PEEK is gradually utilized in fabricating removable partial dentures with the integration of CAD-CAM technology [13]. Removable partial dentures can be fabricated with the integration of the clasp, denture base, framework, and artificial teeth. Some scholars have discovered that the integrated cutting of PEEK material could circumvent issues of stress concentration and the propensity for easy fractures at the joints between different materials observed in traditional cast dentures [4,14]. For patients with anterior tooth isolated loss, artificial teeth often break off repeatedly due to the large bite force of the patient’s anterior teeth, which brings many troubles to the patient. With the use of PEEK material, the removable partial dentures made by integrated cutting may avoid the problem. Some clinical reports underscored CAD-CAM’s effectiveness in crafting PEEK frameworks, presenting them as viable alternatives to traditional removable partial dentures [3,13]. The study applied milled PEEK to fabricate removable maxillary obturator prosthesis with the CAD-CAM technology [9]. The research showed that the PEEK framework produced lower stress on the periodontal ligament but higher stress on the free soft tissue and alveolar bone compared with other materials [10]. In addition, the research results indicated that the PEEK framework had fine stress distribution and reduced the strain around abutments and alveolar ridges compared with metal materials [15]. These findings, coupled with positive clinical feedback, have led to increased patient satisfaction, highlighting the role of PEEK in improving the quality and appeal of removable partial dentures.

Suitability is considered a role criterion for evaluating the clinical feasibility of the RPD framework [16]. There are primarily two manufacturing methods for PEEK removable partial dentures: one is directly milling the PEEK billet, and the other is using resin printing combined with the lost wax technique and filled with PEEK [4,16]. The research demonstrated that the removable partial dentures obtained by direct manufacturing technology achieved better accuracy than the removable partial dentures manufactured by indirect manufacturing technology. At the same time, both methods could satisfy the clinical application standards [17,18]. The study of Ye et al. compared the difference in the suitability of PEEK RPD designed and manufactured by CAD-CAM technology and traditional RPD in vitro [19]. The results indicated that the integrated PEEK RPD had better suitability (overall deviation of 42.8 ± 29.4 μm) in the field of the base, large connector, and composite support compared with traditional cast metal RPD.

Despite these advances, there remains a scarcity of experimental studies on the clinical performance of PEEK RPDs, particularly quantitative analyses comparing them with traditional metal RPDs [20]. Due to the lack of systematic research, it is still challenging to obtain accurate results to evaluate the clinical effect of removable partial dentures fabricated by CAD-CAM technology. Consequently, the examination of clinical properties such as fit and retention for PEEK RPDs warrants more systematic investigation.

In response to this need, this study designed and fabricated the PEEK RPD framework with four types of different teeth defects with CAD-CAM technology to explore the feasibility of digital fabrication of PEEK RPD. The PEEK RPD framework was fabricated by the CAD-CAM technology, while the Co-Cr RPD framework was made by traditional casting. The suitability of the PEEK RPD framework and Co-Cr RPD framework was evaluated by silicone rubber film slice measurement and the three-dimensional image overlay method in vitro. The trueness of the PEEK RPD framework was detected by the three-dimensional image overlay method in vitro. The null hypotheses were that the suitability of the PEEK RPD framework would have no significance with the Co-Cr RPD framework by silicone rubber film slice measurement and three-dimensional image overlay method and that the trueness of the PEEK RPD framework would be within the clinically acceptable error range.

## 2. Materials and Methods

### 2.1. Materials and Groups

The study was divided into two groups: the PEEK group (the RPD framework fabricated by PEEK, HL220302, Shanghai Huliang Biomedical Technology Company, Shanghai, China) and the Co-Cr group (the RPD framework fabricated by Co-Cr, Wirobond C+, Bego Company, Bremen, Germany). The RPD frameworks contained four types of different teeth defects: type one: missing teeth of mandibular bilateral terminal free; type two: missing teeth of mandibular bilateral non-terminal free; type three: missing teeth of maxillary bilateral non-terminal free; and type four: missing teeth of maxillary single posterior.

### 2.2. Selection of Denture Defect Models

**Figure 1:**
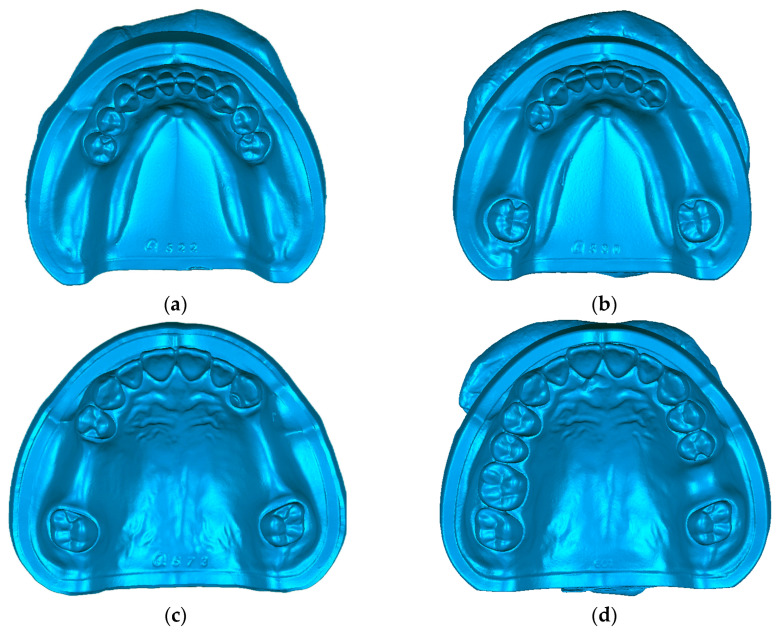
Dentition defect models. (**a**) Bilateral terminal free dentition defects of mandible, (**b**) Bilateral non-terminal free dentition defects of mandible, (**c**) Bilateral non-terminal free dentition defects of maxilla, (**d**) Single posterior dentition defects of maxilla.

We selected four types of dentition defects as the standard oral models (E50HD, NISSIN, Japan). Type one: missing teeth of mandibular bilateral terminal free (36, 37, 46, 47 missing). Type two: missing teeth of mandibular bilateral non-terminal free (35, 36, 44, 45, 46 missing). Type three: missing teeth of maxillary bilateral non-terminal free (15, 16, 24, 25, 26 missing). Type four: missing teeth of maxillary single posterior (26 missing) (Figure 1). The models were scanned by the three-dimensional scanner (D800 3D Scanner, 3-Shape, Copenhagen, Denmark) to acquire the digital data.

### 2.3. Design and Fabrication of the RPD Framework

**Figure 2:**
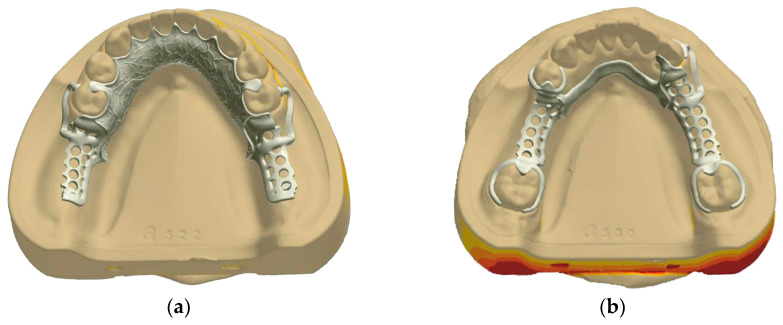

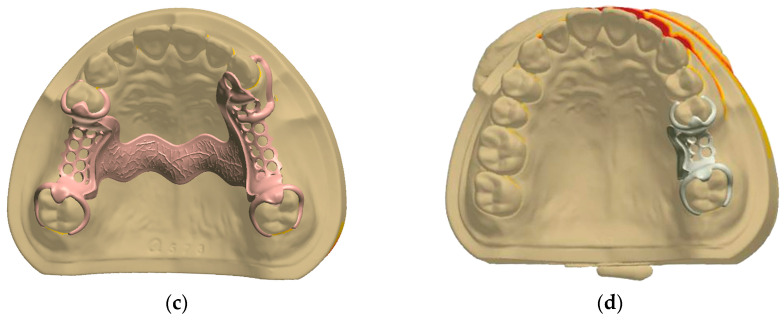
Digital design of PEEK RPD framework. (**a**) Bilateral terminal free dentition defects of mandible, (**b**) Bilateral non-terminal free dentition defects of mandible, (**c**) Bilateral non-terminal free dentition defects of maxilla, (**d**) Single posterior dentition defects of maxilla.

The designs of RPD frameworks were developed using 3Shape dental design software (3Shape Dental System 2018, 3Shape, København, Denmark) (Figure 2). For the PEEK frameworks, fabrication was performed using CAD-CAM technology, while the Co-Cr alloy frameworks were manufactured by the traditional casting process.

The clasp and major connector of the four different types of denture defect models were designed in 3Shape Dental System 2018. Firstly, the initial phase of the design focused on the detailed crafting of clasps and occlusal rests of the corresponding abutment teeth. As the denture defect differed, the design of the clasp and occlusal rest was diversified. The path of insertion for the clasps was precisely aligned with the guide plane, ensuring an optimal depth into the undercut of 0.25 mm. This meticulous attention to detail was applied to both materials, with PEEK clasps designed to have a thickness and width of approximately 1.5 mm and 3.0 mm with the strategic width-to-thickness ratio of 2:1. Meanwhile, Co-Cr clasps were conceived with slightly slimmer dimensions of approximately 1.0 mm in thickness and 2.0 mm in width, also maintaining a strategic width-to-thickness ratio of 2:1. The terminal third of the retaining arm of the clasp effectively engaged with the undercut of the abutment teeth, providing optimal retention. Then, the design process advanced to the specification of major connectors, with considerations made for the diversity of denture defects. The major connector thickness for the PEEK RPD frameworks was established at 2 mm. The thickness of the major connector for the Co-Cr RPD frameworks was set at 0.5 mm. Finally, the design integrated the major connector, the clasps, and the occlusal rests to complete the RPD framework, and the CAD files of the PEEK and Co-Cr RPD design were then exported for fabrication.

The PEEK RPD framework was fabricated by the five-axis cutting machine (ARUM-5X-200, ARUM, Daejeon, Republic of Korea), ensuring precise and integrated cutting from the design data for each denture defect type. The completed PEEK framework design data of different types of denture defects was imported to the five-axis cutting machine and integrated cutting to obtain the PEEK RPD framework.

The design data of the Co-Cr RPD framework was used to print the wax type. The Co-Cr RPD frameworks underwent a traditional fabrication by embedding and casting using the lost wax method. This process utilized a medium-frequency casting machine (HDZP-IV, HaiDeHaoTian, Tianjin, China) and was completed with a final phase of sandblasting using alumina particles (50 μm in diameter) under 500 kPa of air pressure to refine the surface.

### 2.4. The Suitability of RPD Framework by Silicone Rubber Film Slice Measurement

The RPD framework was installed entirely on the model after the light silicone rubber (Honigum, DMG, Hamburg, Germany) was evenly coated on the structure surface of the framework. A vertical pressure of about 20 N was applied to the center of the framework mechanically for 10 min until the silicone rubber was completely solidified. Then, the excess light silicone rubber was removed, and the framework covered with light silicone rubber was split from the model. A layer of heavy silicone rubber was added underneath the light silicone rubber to provide additional support. After the heavy silicone rubber solidified, the silicone rubber was separated from the framework.

**Figure 3:**
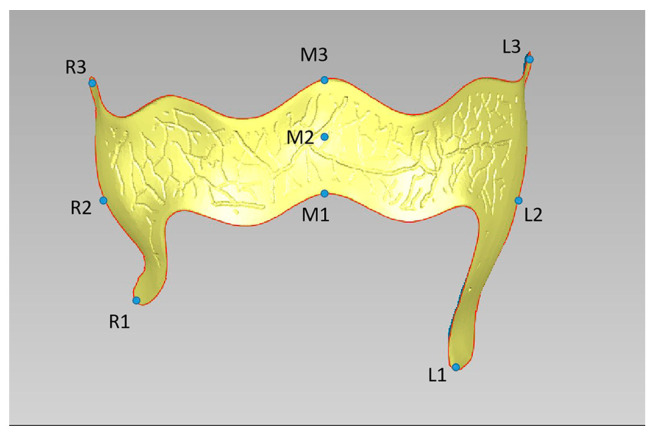
Nine detection sites. L1, L2, L3: Three reference points on the left edge of the major connector; M1, M2, M3: Three reference points in the center of the major connector; R1, R2, R3: Three reference points on the right edge of the major connector.

For analytical precision, nine reference points were established by selecting three locations at each detection site: the left edge, the right edge, and the center of the major connector (Figure 3). The thickness of the light silicone rubber at these nine points was meticulously observed and measured under a stereomicroscope at 30× magnification. The average thickness of the light silicone rubber across these points was computed, establishing an objective measure of the suitability of the framework. This average thickness served as a critical benchmark, reflecting the uniformity of the fit of the RPD framework and adaptation to the surface of the model.

### 2.5. The Suitability of RPD Framework by Three-Dimensional Image Overlay Method

The digital data of the PEEK and Co-Cr RPD framework with or without light silicone rubber were scanned by a high-precision tabletop scanner (d-Station 3D scanner, Breuckmann, Denmark) in this study. The scanner was calibrated before the procedure of scanning. The light silicone rubber was evenly applied to the tissue surface of the framework. Once the silicone rubber solidified completely, any excess silicone rubber was carefully removed from the framework. Subsequently, the framework coated with silicone rubber was detached from the model.

The framework coated with silicone rubber was then positioned on a base and dusted with spray powder (DPT-5, Xinmeida, Shanghai, China) to improve scan visibility. A precision scanner was utilized to capture the framework data with the silicone rubber coating. After scanning, the light silicone rubber was removed from the surface of the framework without altering its position. The framework without silicone rubber was sprayed with the powder. The scanning proceeded again to acquire the data of the framework without silicone rubber.

**Figure 4:**
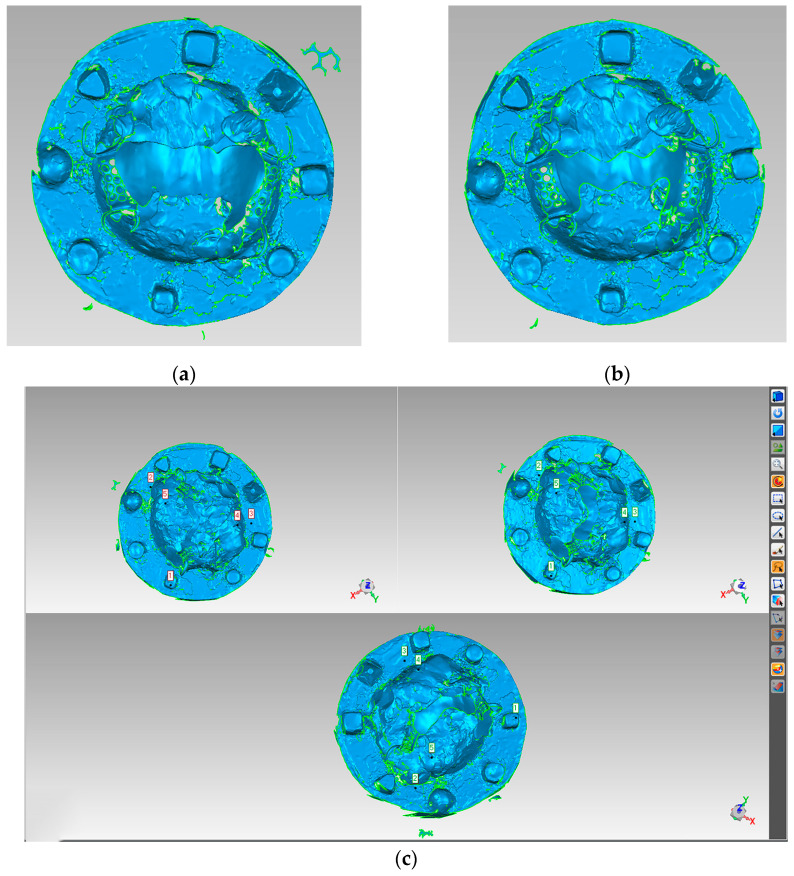
(**a**) Scanning data of framework with light silicone rubber, (**b**) Scanning data of framework without light silicone rubber, (**c**) Manual registered alignment of two scanning data in reverse engineering software.

The image analysis software (Geomagic Wrap 2017, Geomagic, Triangle Park, NC, USA) (Figure 4a,b) was employed to manually register and align the scanned data of the frameworks, both with and without light silicone rubber (Figure 4c). The registered matching images were analyzed for deviation. The calculated deviation represented the thickness of the light silicone rubber.

### 2.6. The Trueness of the PEEK RPD Framework with CAD-CAM Technology

The PEEK RPD frameworks were scanned with spray powder by the high-precision tabletop scanner. The image analysis software (Geomagic Wrap 2017, Geomagic, USA) was used to compare the data acquired by the PEEK RPD framework scanning and the image data with CAD design. The registered matching images provided a deviation analysis to evaluate the accuracy of the PEEK RPD framework with CAD-CAM technology.

### 2.7. Statistical Analysis

SPSS 23 statistical analysis software was used to analyze the above-measured values. Two independent sample *t*-tests were conducted to compare the PEEK and the Co-Cr RPD frameworks within each of the four distinct types of denture defects.

## 3. Results

### 3.1. The Suitability of Silicone Rubber Film Slice Measurement

The suitability values of the PEEK group were 88.37–197.12 μm, compared to 135.06–239.20 μm for the Co-Cr group (Table 1). In type one, the thickness values of the light silicone rubber of the PEEK group were 162.16 ± 23.45 μm, and those of the Co-Cr group were 224.27 ± 25.27 μm. In type two, the thickness values were 88.37 ± 23.61 μm for the PEEK group and 135.06 ± 17.01 μm for the Co-Cr group. In type three, the PEEK group thickness values were 197.12 ± 16.77 μm, while those of the Co-Cr group were about 228.90 ± 29.19 μm. In type four, the PEEK and the Co-Cr groups displayed thickness values of 158.48 ± 44.35 μm and 239.20 ± 16.36 μm. The thickness values of the PEEK group were lower than those of the Co-Cr group in four types.

Statistical analysis by independent sample *t*-tests was conducted to determine the suitability of different materials for each group of the RPD framework. The results displayed that the differences in suitability were statistically significant between the PEEK group and the Co-Cr group in type one, type two, and type four (*p* < 0.05). The suitability of the RPD framework of the PEEK group was superior to that of the Co-Cr group.

### 3.2. The Suitability of Three-Dimensional Image Overlay Method

Suitability measurements obtained through the three-dimensional image overlay method were detailed in Table 2. The suitability values of the PEEK group from type one to type four were 377.96 ± 46.49 μm, 398.14 ± 35.06 μm, 277.90 ± 6.39 μm, and 400.68 ± 63.59 μm. The suitability values of the Co-Cr group from type one to type four were 323.90 ± 25.58 μm, 404.06 ± 60.65 μm, 246.68 ± 43.34 μm, and 331.64 ± 24.15 μm. The results exhibited that the *p* values of the suitability in four types were higher than 0.05 between the PEEK group and the Co-Cr group by the application of the independent sample *t*-test. There were no statistical differences in the suitability of the two groups measured by the three-dimensional image overlay method.

**Figure 5:**
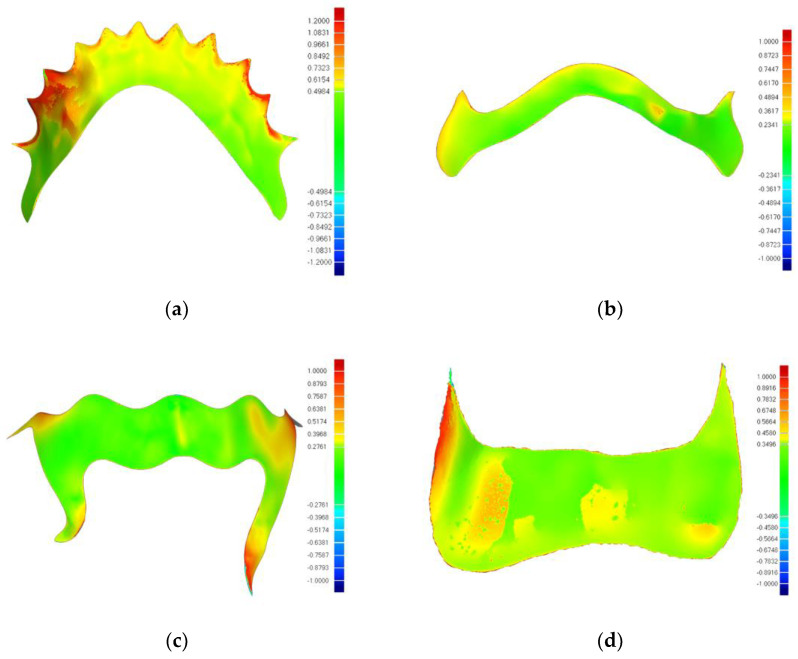
The color-coded maps of suitability of PEEK frameworks. (**a**) Bilateral terminal free dentition defects of mandible, (**b**) Bilateral non-terminal free dentition defects of mandible, (**c**) Bilateral non-terminal free dentition defects of maxilla, (**d**) Single posterior dentition defects of maxilla.

The deviation analysis chromatogram of the PEEK group was analyzed in Figure 5. The deviation of the framework edge (including the gingival margin) was generally minimal but scattered in the high deviation area. The high-deviation area was intermittently distributed, with some reaching nearly 1000 μm. The central region of the framework exhibited a relatively larger high-deviation zone, with deviation values ranging from approximately 400–500 μm. In type one (missing teeth of mandibular bilateral terminal free) of the PEEK group, the deviations were predominantly localized near the gingival margin, reaching up to 1000 μm in particular areas.

### 3.3. The Trueness of the PEEK RPD Framework with CAD-CAM Technology

**Figure 6:**
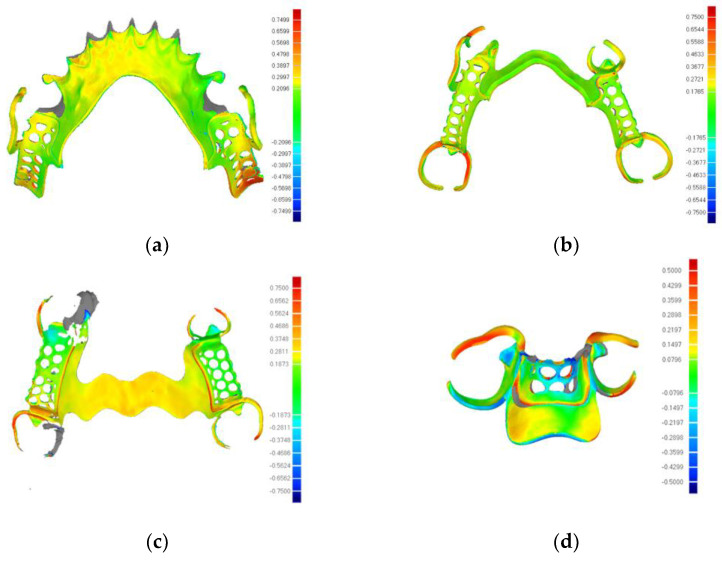
The color-coded maps of trueness of PEEK framework major connectors (**a**–**d**).

The trueness values of the PEEK RPD framework, as depicted in Table 3, were 196.86 ± 23.55 μm in type one, 169.92 ± 10.91 μm in type two, 256.08 ± 15.35 μm in type three, and 180.34 ± 1.27 μm in type four. Figure 6 illustrates the color-coded maps of the trueness of the RPD framework measured by the three-dimensional image overlay method. The error values of each group were in the range of 50–426.3 μm [21]. The trueness of the PEEK RPD framework was within the acceptable margins for prosthesis processing.

## 4. Discussion

Suitability and trueness are pivotal factors in evaluating the performance of RPD frameworks fabricated using digital technologies [22]. The aim of the study was to evaluate the suitability and trueness of the removable partial denture (RPD) framework fabricated by PEEK with CAD-CAM technology and explore the feasibility of digital fabrication of the PEEK RPD framework. The results indicated that there were significant differences in suitability between the PEEK and Co-Cr groups for types one, two, and four measured by silicone rubber film slice (*p* < 0.05). The suitability values by the three-dimensional image superposition method showed no statistical differences (*p* > 0.05) in four types between the two groups. Thus, the hypothesis of the study that the suitability of the PEEK RPD framework would have no difference with the Co-Cr RPD framework by silicone rubber film slice measurement and the three-dimensional image overlay method was rejected. Moreover, the trueness values of the PEEK RPD framework, ranging from 50 to 426.3 μm, were within the allowable range of clinical error. Therefore, the research hypothesis that the trueness of the PEEK RPD framework would be within the clinically acceptable range of error was accepted.

Suitability stands as a critical metric in the clinical performance evaluation of RPD frameworks, reflecting their fit, comfort, and functionality within the oral environment. Despite its significance, a universally accepted standard for measuring suitability is still lacking [20]. Since the 1980s, researchers have employed the silicone rubber film replication method to quantitatively measure the gap between RPD frameworks and oral soft and hard tissues, thereby assessing the suitability of the framework. This method encompassed several techniques for measuring the thickness of the silicone rubber film, such as the silicone rubber film slice measurement, the three-dimensional analysis of gypsum models, and the three-dimensional analysis of recycled models [23]. However, there was no standard measurement point for the silicone rubber film slice measurement method, leading researchers to choose varying numbers and locations of points based on the specific goals of their studies [24,25].

In the study, the silicone rubber film slice measurement method and three-dimensional image overlay method were conducted to detect the suitability of RPD frameworks in different types of dentition defects. The three-dimensional image overlay method was chosen to calculate the average fitness of the whole framework, and the deviation analysis chromatogram was observed to evaluate the fitness differences of diverse parts of the framework. The measurement points were primarily chosen along the edge of the major connector, including the gingival margin, to gauge the fitness of the framework for the silicone rubber film slice measurement method. The three-dimensional image overlay method was chosen to calculate the average fitness of the whole framework for a holistic assessment, with a deviation analysis chromatogram providing insight into the differences in the fitness among diverse framework sections. The suitability values of the PEEK group were measured between 88.37–197.12 μm, and those of the Co-Cr group were measured between 135.06–239.20 μm in the silicone rubber film slice measurement method. The suitability values of the PEEK group were between 277.90–400.68 μm, and that of the Co-Cr group was between 246.68–404.06 μm in the three-dimensional image overlay method.

In the realm of prosthetic dentistry, particularly concerning the fabrication and application of removable partial dentures, the suitability of the RPD framework spanning from 50 to 311 μm was clinically acceptable [26]. Arnold et al. discovered that the suitability of a traditional cast metal framework could reach 133 ± 59 μm in the horizontal direction and 73 ± 25 μm in the vertical direction [22]. The study of Soltanzadeh et al. used traditional wax loss technology and CAD-CAM technology to manufacture metal RPD frameworks for missing teeth in the maxillary (the third type of Kennedy classification). The experiment detected and analyzed the suitability of large connectors, clings, and other parts [26]. The analysis showed that the traditional RPD framework had the best suitability (deviation 27 ± 40 μm), and the CAD-CAM framework had a slightly lower suitability (150 ± 13 μm), both of which successfully met the clinical requirements. Ye et al. carried out a quantitative analysis on the suitability of an integrated PEEK RPD, designed and fabricated by CAD-CAM technology, measuring the base, large connector, and overall suitability. The integrated RPD of PEEK (overall deviation 42.8 ± 29.4 μm) showed superior suitability compared with the traditional cast metal RPD [19]. The culmination of findings from various studies evidenced that the advanced integrated RPD of PEEK designed and manufactured by CAD-CAM technology did not merely approximate but potentially surpassed the performance metrics in suitability.

In the experiment, measurement points were strategically chosen along the edge of the framework, with nine reference points established by selecting three locations at each detection site: the left edge, the right edge, and the center of the major connector. This selection, while perhaps not encompassing the complete suitability of the framework, provided a reference for the edge tightness of the framework. The difference in suitability between the two methods might be related to the fact that the measurement points selected by the silicone rubber film slice measurement method were mainly located at the edge of the framework, which reflected that the edge tightness was fine. We detected that the edge deviation value of the framework was generally low, and the high deviation area was primarily located in the center of the framework with the deviation analysis chromatogram. This was consistent with the result that, as mentioned above, the suitability measured by the silicone rubber film slice measurement method was lower than that measured by the three-dimensional image overlay method. Furthermore, increased deviations were distributed near the gingival margin in the type one dentition defect, potentially due to reduced adhesion at the gingival papilla in the standard model and a gap between the tongue tip and gum after the framework was inserted. It could be concluded that the edge adhesion of the PEEK framework was significantly higher than that of the Co-Cr framework (*p* < 0.05) according to the silicone rubber film slice measurement. Meanwhile, the results of the three-dimensional image overlay method showed no statistical difference in suitability between the PEEK and the Co-Cr group in four types. In combination with previous research, it was considered that the suitability of the PEEK framework could meet the needs of clinical application [26,27].

The trueness of the fabricated PEEK framework indicated that it was acceptable and controllable in the study. The error values were within the permissible range of prosthesis processing (50–426.3 μm) [21]. Tasaka et al. studied the trueness of RPD frameworks manufactured by two methods: one is the 3D printing technology and casting (AM-Cast), and the other is the selective laser sintering (SLS) method [28]. The researchers reported that the variations in trueness of the frameworks between AM-Cast and SLS ranged from −185 ± 138 to 352 ± 143 μm and −166 ± 9 to 123 ± 9 μm. The trueness of Co-Cr and Ti-6Al-4V alloy frameworks for RPDs fabricated by selective laser melting underwent evaluation in the research of Peng et al. [29]. The results indicated that the difference values of trueness ranged from 323 μm to 550 μm, which was considered to achieve trueness, was as high as those of traditional casting methods. The systematic review of Ana et al. evaluated seven articles that complied with the requirements for inclusion [15]. Due to the misfits and mismatches being beneath the acceptable clinical threshold for RPDs, the results pointed out the accuracy of the digital technique for RPD frameworks. PEEK showed a better fit than traditional metal cast RPDs. They considered a gap from 50 to 311 mm as a clinically acceptable fit of the RPD framework. Based on previous research, the trueness of the PEEK framework in this study, varying from 169.92 ± 10.91 μm to 256.08 ± 15.35 μm was within the clinically acceptable range. The CAD designs for prostheses of four types of teeth defects could be satisfactorily copied into the PEEK framework. Although the comprehensive structure of the framework suffered some slight alterations during the processing, these modifications did not affect the final PEEK framework, which met the existing clinical requirements for the trueness of the removable partial denture [30].

Varying designs of removable partial dentures, involving variances in the shape of the clasp and thickness of the framework, had been shown to possess a significant effect on abutment teeth as well as the surrounding oral soft and hard tissues [31,32,33,34]. With the goal of clarifying the design principles of PEEK RPDs, the stress distribution of the PEEK RPD framework in different designs will be further improved and analyzed in the subsequent research to find the best design for various cases of dentition defect. Combined with clinical research, it was possible for the integrated PEEK RPD with digital technology to be applied in prosthodontics.

With the limitation of the study, this research of the PEEK PRD framework was an in vitro study. The in vitro experiments could not fully simulate the actual situation in the mouth. The selection of denture defect models in the survey was considered based on the span range of the framework and the difference between mandibular and maxillary. Combining the above considerations, four different types of denture defect models were designed and fabricated for the RPD framework. The PEEK PRD framework design and evaluation according to the Kennedy classification and other factors will be considered for further study. At the same time, further in vivo studies of the PEEK PRD framework need to be explored to provide a basis for the clinical application of PEEK material.

## 5. Conclusions

In the study, the suitability values of the PEEK group by silicone rubber film slice measurement were lower than the Co-Cr group in four types. There were statistical differences between the PEEK and the Co-Cr groups in type one, type two, and type four. The suitability values of the PEEK and Co-Cr groups by the three-dimensional image overlay method showed no statistical differences in the four types. The trueness values of the PEEK group with four types were within the allowable range of clinical error.

The suitability and trueness of the PEEK RPD framework fabricated by CAD-CAM technology both met the demand of the clinical prosthesis. The digital fabrication of PEEK had great potential in removable partial dentures.

## Figures and Tables

**Table 1:** Comparison of the suitability of frameworks with different materials by silicone rubber film slice measurement (μm).

Type	1	2	3	4
PEEK (*n* = 5)	162.16 ± 23.45	88.37 ± 23.61	197.12 ± 16.77	158.48 ± 44.35
Co-Cr (*n* = 5)	224.27 ± 25.27	135.06 ± 17.01	228.90 ± 29.19	239.20 ± 16.36
*p* value	0.004	0.007	0.068	0.012

**Table 2:** Comparison of the suitability of frameworks with different materials by three-dimensional image superposition method (μm).

Type	1	2	3	4
PEEK (*n* = 5)	377.96 ± 46.49	398.14 ± 35.06	277.90 ± 6.39	400.68 ± 63.59
Co-Cr (*n* = 5)	323.90 ± 25.58	404.06 ± 60.65	246.68 ± 43.34	331.64 ± 24.15
*p* value	0.052	0.855	0.150	0.053

**Table 3:** Trueness of PEEK frameworks by CAD-CAM technology (μm).

Type	1	2	3	4
PEEK (*n* = 5)	231.5	158.4	243.7	181.2
	210.9	185.9	281.4	178.9
	176.2	164.6	247.4	181.3
	183.7	164.9	248.3	179.0
	182.0	175.8	259.6	181.3
Mean	196.86	169.92	256.08	180.34
SD	23.55	10.91	15.35	1.27

## Data Availability

The data presented in this study will be made available on request (due to privacy).

## References

[1] Revilla-León M., Gómez-Polo M., Vyas S., Barmak A.B., Gallucci G.O., Att W., Özcan M., Krishnamurthy V.R. (2023). Artificial Intelligence Models for Tooth-Supported Fixed and Removable Prosthodontics: A Systematic Review. J. Prosthet. Dent..

[2] Almufleh B., Arellanob A., Tamimi F. (2023). Patient-Reported Outcomes and Framework Fit Accuracy of Removable Partial Dentures Fabricated Using Digital Techniques: A Systematic Review and Meta-Analysis. J. Prosthodont..

[3] Takaichi A., Fueki K., Murakami N., Ueno T., Inamochi Y., Wada J., Arai Y., Wakabayashi N. (2022). A Systematic Review of Digital Removable Partial Dentures. Part II: CAD/CAM Framework, Artificial Teeth, and Denture Base. J. Prosthodont. Res..

[4] Sadek S.A. (2019). Comparative Study Clarifying the Usage of PEEK as Suitable Material to Be Used as Partial Denture Attachment and Framework. Open Access Maced. J. Med. Sci..

[5] Najeeb S., Zafar M.S., Khurshid Z., Siddiqui F. (2016). Applications of Polyetheretherketone (PEEK) in Oral Implantology and Prosthodontics. J. Prosthodont. Res..

[6] Panayotov I.V., Orti V., Cuisinier F., Yachouh J. (2016). Polyetheretherketone (PEEK) for Medical Applications. J. Mater. Sci. Mater. Med..

[7] Andrikopoulou E., Zoidis P., Artopoulou I.-I., Doukoudakis A. (2016). Modified PEEK Resin Bonded Fixed Dental Prosthesis for a Young Cleft Lip and Palate Patient. J. Esthet. Restor. Dent..

[8] Stawarczyk B., Beuer F., Wimmer T., Jahn D., Sener B., Roos M., Schmidlin P.R. (2013). Polyetheretherketone-a Suitable Material for Fixed Dental Prostheses?. J. Biomed. Mater. Res. B Appl. Biomater..

[9] Costa-Palau S., Torrents-Nicolas J., Brufau-de Barberà M., Cabratosa-Termes J. (2014). Use of Polyetheretherketone in the Fabrication of a Maxillary Obturator Prosthesis: A Clinical Report. J. Prosthet. Dent..

[10] Chen X., Mao B., Zhu Z., Yu J., Lu Y., Zhang Q., Yue L., Yu H. (2019). A Three-Dimensional Finite Element Analysis of Mechanical Function for 4 Removable Partial Denture Designs with 3 Framework Materials: CoCr, Ti-6Al-4V Alloy and PEEK. Sci. Rep..

[11] Akl M.A., Stendahl C.G. (2022). Removable Partial Denture Frameworks in the Age of Digital Dentistry: A Review of the Literature. Prosthesis.

[12] Liu Y., Fang M., Zhao R., Liu H., Li K., Tian M., Niu L., Xie R., Bai S. (2022). Clinical Applications of Polyetheretherketone in Removable Dental Prostheses: Accuracy, Characteristics, and Performance. Polymers.

[13] Harb I.E., Abdel-Khalek E.A., Hegazy S.A. (2019). CAD/CAM Constructed Poly(Etheretherketone) (PEEK) Framework of Kennedy Class I Removable Partial Denture: A Clinical Report. J. Prosthodont..

[14] Zoidis P., Papathanasiou I., Polyzois G. (2016). The Use of a Modified Poly-Ether-Ether-Ketone (PEEK) as an Alternative Framework Material for Removable Dental Prostheses. A Clinical Report. J. Prosthodont..

[15] Carneiro Pereira A.L., Bezerra de Medeiros A.K., de Sousa Santos K., Oliveira de Almeida É., Seabra Barbosa G.A., da Fonte Porto Carreiro A. (2021). Accuracy of CAD-CAM Systems for Removable Partial Denture Framework Fabrication: A Systematic Review. J. Prosthet. Dent..

[16] Ahmed K.E. (2018). We’re Going Digital: The Current State of CAD/CAM Dentistry in Prosthodontics. Prim. Dent. J..

[17] Papathanasiou I., Kamposiora P., Papavasiliou G., Ferrari M. (2020). The Use of PEEK in Digital Prosthodontics: A Narrative Review. BMC Oral Health.

[18] Bathala L., Majeti V., Rachuri N., Singh N., Gedela S. (2019). The Role of Polyether Ether Ketone (Peek) in Dentistry—A Review. J. Med. Life.

[19] Ye H., Li X., Wang G., Kang J., Liu Y., Sun Y., Zhou Y. (2018). A Novel Computer-Aided Design/Computer-Assisted Manufacture Method for One-Piece Removable Partial Denture and Evaluation of Fit. Int. J. Prosthodont..

[20] Yan X., Lin H., Wu Y., Bai W. (2018). Effect of Two Heat Treatments on Mechanical Properties of Selective-Laser-Melted Co-Cr Metal-Ceramic Alloys for Application in Thin Removable Partial Dentures. J. Prosthet. Dent..

[21] Negm E.E., Aboutaleb F.A., Alam-Eldein A.M. (2019). Virtual Evaluation of the Accuracy of Fit and Trueness in Maxillary Poly(Etheretherketone) Removable Partial Denture Frameworks Fabricated by Direct and Indirect CAD/CAM Techniques. J. Prosthodont..

[22] Arnold C., Hey J., Schweyen R., Setz J.M. (2018). Accuracy of CAD-CAM-Fabricated Removable Partial Dentures. J. Prosthet. Dent..

[23] Ahmed N., Abbasi M.S., Haider S., Ahmed N., Habib S.R., Altamash S., Zafar M.S., Alam M.K. (2021). Fit Accuracy of Removable Partial Denture Frameworks Fabricated with CAD/CAM, Rapid Prototyping, and Conventional Techniques: A Systematic Review. Biomed. Res. Int..

[24] Ye H., Ning J., Li M., Niu L., Yang J., Sun Y., Zhou Y. (2017). Preliminary Clinical Application of Removable Partial Denture Frameworks Fabricated Using Computer-Aided Design and Rapid Prototyping Techniques. Int. J. Prosthodont..

[25] Lee J.-W., Park J.-M., Park E.-J., Heo S.-J., Koak J.-Y., Kim S.-K. (2017). Accuracy of a Digital Removable Partial Denture Fabricated by Casting a Rapid Prototyped Pattern: A Clinical Study. J. Prosthet. Dent..

[26] Soltanzadeh P., Suprono M.S., Kattadiyil M.T., Goodacre C., Gregorius W. (2019). An In Vitro Investigation of Accuracy and Fit of Conventional and CAD/CAM Removable Partial Denture Frameworks. J. Prosthodont..

[27] Oh K.C., Yun B.S., Kim J.-H. (2022). Accuracy of Metal 3D Printed Frameworks for Removable Partial Dentures Evaluated by Digital Superimposition. Dent. Mater..

[28] Tasaka A., Shimizu T., Kato Y., Okano H., Ida Y., Higuchi S., Yamashita S. (2020). Accuracy of Removable Partial Denture Framework Fabricated by Casting with a 3D Printed Pattern and Selective Laser Sintering. J. Prosthodont. Res..

[29] Peng P.-W., Hsu C.-Y., Huang H.-Y., Chao J.-C., Lee W.-F. (2022). Trueness of Removable Partial Denture Frameworks Additively Manufactured with Selective Laser Melting. J. Prosthet. Dent..

[30] Souza Curinga M.R., Claudino Ribeiro A.K., de Moraes S.L.D., do Egito Vasconcelos B.C., da Fonte Porto Carreiro A., Pellizzer E.P. (2023). Mechanical Properties and Accuracy of Removable Partial Denture Frameworks Fabricated by Digital and Conventional Techniques: A Systematic Review. J. Prosthet. Dent..

[31] Trivedi S. (2014). Finite Element Analysis: A Boon to Dentistry. J. Oral Biol. Craniofac. Res..

[32] Mousa M., Jamayet N., Lynch E., Husein A. (2020). Biomechanical Stress in Removable Complete Dental Prostheses: A Narrative Review of Finite Element Studies. J. Int. Oral Health.

[33] Mousa M.A., Abdullah J.Y., Jamayet N.B., Alam M.K., Husein A. (2021). Biomechanical Stress in Obturator Prostheses: A Systematic Review of Finite Element Studies. Biomed. Res. Int..

[34] Zarrati S., Bahrami M., Heidari F., Kashani J. (2015). Three Dimensional Finite Element Analysis of Distal Abutment Stresses of Removable Partial Dentures with Different Retainer Designs. J. Dent..

